# Implementation of an Education Value Unit (EVU) System to Recognize Faculty Contributions

**DOI:** 10.5811/westjem.2015.8.26136

**Published:** 2015-11-12

**Authors:** Joseph House, Sally A. Santen, Michele Carney, Michele Nypaver, Jonathan P. Fischer, Laura R. Hopson

**Affiliations:** * University of Michigan Medical School, Department of Emergency Medicine Children’s Emergency Services, Ann Arbor, Michigan; † University of Michigan Medical School, Department of Emergency Medicine, Department of Learning Health Sciences, Ann Arbor, Michigan; ‡ University of Michigan, School of Public Health, Ann Arbor, Michigan; § University of Michigan Medical School, Department of Emergency Medicine, Ann Arbor, Michigan

## Abstract

**Introduction:**

Faculty educational contributions are hard to quantify, but in an era of limited resources it is essential to link funding with effort. The purpose of this study was to determine the feasibility of an educational value unit (EVU) system in an academic emergency department and to examine its effect on faculty behavior, particularly on conference attendance and completion of trainee evaluations.

**Methods:**

A taskforce representing education, research, and clinical missions was convened to develop a method of incentivizing productivity for an academic emergency medicine faculty. Domains of educational contributions were defined and assigned a value based on time expended. A 30-hour EVU threshold for achievement was aligned with departmental goals. Targets included educational presentations, completion of trainee evaluations and attendance at didactic conferences. We analyzed comparisons of performance during the year preceding and after implementation.

**Results:**

Faculty (N=50) attended significantly more didactic conferences (22.7 hours v. 34.5 hours, p<0.005) and completed more trainee evaluations (5.9 v. 8.8 months, p<0.005). During the pre-implementation year, 84% (42/50) met the 30-hour threshold with 94% (47/50) meeting post-implementation (p=0.11). Mean total EVUs increased significantly (94.4 hours v. 109.8 hours, p=0.04) resulting from increased conference attendance and evaluation completion without a change in other categories.

**Conclusion:**

In a busy academic department there are many work allocation pressures. An EVU system integrated with an incentive structure to recognize faculty contributions increases the importance of educational responsibilities. We propose an EVU model that could be implemented and adjusted for differing departmental priorities at other academic departments.

## INTRODUCTION

Changes in healthcare have placed pressure on emergency departments (EDs). For academic EDs, this presents added challenges as they struggle to balance their tripartite missions of clinical care, research, and education. There are often fewer incentives for educational activities than for other domains, and as a result, education may be given a lower priority.^5^

Efforts have been made to better align departmental budgets between clinical care, research, and education. “Mission-based budgeting” began in 1999 and has grown in popularity. 1, 2 While this system allocated more resources to educational activity, departments struggled to equitably distribute these funds to individual faculty. The result was often that incentives were not tied to specific education-related activity, but were evenly distributed among faculty. 2 In response, medical schools attempted to quantify educational activity using the relative value unit system of measuring patient care activity as a model. 2–5 The educational value unit (EVU), although promising in its potential to incentivize educational activity, has not achieved widespread utilization or been studied extensively. This is especially true in the ED, where only one study was published a decade ago. 5

In 2011, our chair established a Faculty Incentive Task Force that included faculty representing all departmental missions within our academic ED. Faculty contribution to the educational mission was identified as a core metric, and an educational subcommittee was formed to review the available literature and other departmental practices to develop measurement criteria. The purpose of this study was to determine the feasibility of an EVU system in an academic ED and to examine its effect on faculty behavior in the educational mission.

## METHODS

### Study Design and Setting

This is a prospective observational study that was reviewed by the IRB and determined to be exempt.

### Methods and Measurements

Through group consensus, the Faculty Incentive Task Force agreed upon broad priorities supporting education. These included providing lectures, conference participation, participation in trainee recruitment, and completion of trainee assessments. An analysis of all educational activity performed during the prior academic year was performed, which allowed identification of common activities as well as creation of a tracking process.

Four main educational activity categories for EVUs (measured in hours) were created for educational contribution to the department. Activities were informed by medical school departmental funding models, and each activity was assigned a standardized time value determined by group consensus (Table 1). In general, preparing and leading an educational session de novo, earned more hours than presenting an existing lecture or assisting in a conference. Value was also given to activities requiring faculty time, including trainee recruitment as well as conference attendance. The “additional” teaching category included educational activities such as teaching in other departments, mentoring, and involvement in educational committees. It relied on faculty self-report. This final category was incorporated during the post-implementation year based on faculty feedback and was excluded from the analysis.

We used administrative data collection to track the four intradepartmental categories. These were tracked from existing materials from the residency, fellowship and student domains, including final conference schedules, attendance records, and Medhub reports. We relied on faculty input to complete the final “additional” teaching category. In addition, we created mechanisms to regularly update faculty as to their participation levels. The first iteration of the EVU calculation was tested against faculty activity from the immediate past academic year, and an initial benchmark selected which would allow the majority of faculty to meet based on existing activities in order to foster support for the program during implementation. The program was implemented in July 2013.

### Outcomes and Analysis

After implementation of the program, we compared impact on educational priority items (e.g. conference attendance, completion of resident evaluations) to pre-implementation levels using paired t-tests. Data analysis was performed using Microsoft Excel 2010 and graphpad.com calculators. We analyzed EVU achievement in total and all individual domains. Completion rates for resident and fellow evaluations by faculty for July 2012–May 2013 (11 pre-implementation months) and July 2013–May 2014 (11 post-Implementation months) were compared. We defined a faculty member as completing evaluations for the month if they completed at least one resident or fellow evaluation during a given month. Faculty were responsible for determining which trainees they were able to evaluate.

## RESULTS

We included in the analysis (N=50) faculty members from the children’s and adult divisions of the ED who were working clinically during both the pre- and post-implementation periods and subject to the incentive program

The differences between the pre-implementation period and the EVU measurement period are presented in Table 2. Faculty attended significantly more didactic conferences and completed more resident monthly evaluations. The majority of faculty members (84%) met the 30-hour threshold for compliance in the pre-implementation year and 94% in the post-implementation year. There was a small but significant increase in total EVUs between the years due to increased conference attendance and evaluation completion. Interestingly, faculty members who tend not to complete resident evaluations did not change with the new system; of the eight faculty members who completed zero resident evaluations in either academic year, five had zero both years. In contrast, faculty members who completed some evaluations tended to do more under the incentive system. There were no differences between the periods in EVUs attained for educational presentations and trainee recruitment.

Overall, administrative time required was estimated at one hour per month in each of the three major domains (student, residency, and fellowships) and approximately 10 hours annually for development and distribution of biannual progress reports to faculty and departmental leadership.

## DISCUSSION

This study demonstrated that a system of applying value to educational activity is feasible. Additionally, data indicate a positive effect on physician participation while providing objective data for reward for effort supporting educational activities. It is worthwhile to note that despite the small increase in overall educational activities (EVUs), those increases were confined to the smaller scale activities such as evaluation completion and conference attendance. One may speculate that the larger time investment involved in creating a conference presentation is not felt worth the investment despite the weighting of hours in the EVU system.

A characteristic of this system that likely contributed to its success is that it relies on multiple sources of motivation to change faculty behavior. The most obvious motivation was a financial incentive. Although it is commonly believed that financial incentives can change behavior, the literature studying the effect of financial incentives on primary care physician behavior has been inconclusive. 6 In the current study, the maximum financial bonus related to educational activities was approximately 1% of annual salary. The EVU represented one component of an eight-part overall ED incentive system representing educational, scholarly, and clinical goals each with an equal weight applied universally to all faculty.

While faculty response to the amount of the bonus was beyond the scope of this study, it is not clear that the financial incentive itself was sufficient to explain all of the results. An additional motivation may also be the desire for high achievement or a Hawthorne-type effect. This system made educational activity an explicit priority. It also included EVUs as part of each physician’s annual review with the chair. As such, achieving high EVUs was more closely associated with desired performance.

The final motivating factor was likely peer encouragement. Participation in some of the educational activities was made public with monthly summaries. As such, there may have been pressure for physicians to attend events to increase the publicly displayed attendance.

The combined effects of these three sources of motivation are what likely brought about our positive results. Interestingly, our results were somewhat mixed as metrics in prioritized subcategories improved, but overall EVUs achieved experienced only a slight increase. This suggests that individuals tended to put their emphasis on the categories that were most easily recognized. Further, there was no increased incentive to go beyond the threshold EVU, although most faculty did. However, it should be noted that participation in all domains was stable or increased, so we were able to cumulatively increase faculty participation.

This study builds on the work of Khan and Simon, who developed a system in 2003 to quantify and reward teaching activity in an ED. 5 Their system applied weights to activities that were deemed more essential, as opposed to our system that was based on the time required to complete each task. While the Khan system included only teaching activities for medical students, our system included a wide array of educational activities to recognize diverse contributions. Although pre-implementation data were not available, Khan and Simon report progressive increases in both group and individual productivity during the first three years of implementation. Our results build on previous literature suggesting that incentive systems may be effective at increasing educational activity in an academic ED.

## LIMITATIONS

There are several limitations to this study. First, it is a single site focused on initial response to the implementation of the EVU system and generalizability may be limited. It would be difficult to implement identical EVU systems across multiple EDs; thus, we encourage others to customize EVU systems and study the results. Second, it is difficult to determine the exact motivations of faculty behaviors. Whether the EVU set the tone for a culture recognizing the importance of education or whether there were other factors at play is not known. Third, we did need to balance administrative feasibility with faculty desire to recognize all possible educational activities. Finally, organizational politics had an effect on the EVU program with the decision to emphasize departmental activities rather than extra-departmental.

## CONCLUSION

As external time and financial pressures continue to increase, it is imperative that academic EDs remain committed to their educational missions. Achieving this will require innovative methods to use limited resources. Once developed, the EVU system may be tailored to address changing departmental priorities and challenges. This study demonstrates that development of an EVU system to incentivize faculty is feasible and effective in motivating faculty to meet educational responsibilities. This study represents an important step in that direction and hopefully will prompt further investigation into how to best promote educational activity in busy academic EDs.

## Figures and Tables

**Table 1:** Education value unit per educational activity.

Departmental activities-baseline	Expected minimums
1) Leading a new educational sessions (including residency or fellowship lectures, administrative track or other departmental seminar, or intern orientation lecture(10 hours of time to account for preparation for each 1 hour presented) Or Preparing & leading an active learning session: (e.g. Skills & procedure labs, simulation, oral board exams/clinical skills exams cases, small group sessions, focused residency retreats, Peds OSCEs (5 hours credit:1 hour presented) Or Assisting with active learning sessions, small groups, skills labs, mentoring resident session, focused mentoring (2 hours credit:1 hour presented) Or Student teaching sessions (1 hour credit:1 hour presented) Or Other teaching activities EMIG, clinical skills assessments (CSA) (1 hour:1 hour)	10 hours
2) Didactic conference attendance (1:1) optimal 3hrs/month=36	10 hours
3) Completion of evaluations of residents & fellows (1 hour per month)	10 hours
4) Recruitment interviews for residency or fellowship programs (1 hour:1 hour)	
5) Additional activities-resident mentoring, educational activities outside department, educational committees (maximum 10 hrs.)	
Total (minimum expected)	Total=30 hrs

*Peds*, pediatrics; *OSCEs*, objective structured clinical examination; *EMIG*, emergency medicine interest group

**Table 2:** Year-to-year comparison of education value unit (EVU) results pre and post implementation. Total EVUs increase as a result of increased activity with the conference attendance and evaluation completion subcategories. Participation in recruitment activities and educational sessions are unchanged. The final category of non-departmental educational activities was in effect only for the post-implementation year so was excluded from the analysis. Domains involved in each subcategory include residency, fellowships, and medical students.

	Total EVUs (hours) attained	EVUs (hours) earned from all departmental educational sessions (R,F,S)	EVUs (hours) earned from didactic conference attendance (R,F)	EVUs (hours) from completion of evaluations (R, F)	EVUs (hours) from educational recruitment activities (R,F)
Pre-implementation year	Mean 94.4 SD 75 Range 0–390	Mean 56.9 SD 52.2 Range 0–239	Mean 22.7 SD 16.8 Range 0–75	Mean 5.9 SD 3.8 Range 0–16	Mean 8.8 SD 19.4 Range 0–12
Post-implementation year	Mean 109.8 SD 90 Range 0–521	Mean 58.5 SD 68.7 Range 0–369	Mean 34.5 SD 19.9 Range 0–109	Mean 8.8 SD 6.4 Range 0–26	Mean 7.9 SD 16.3 Range 0–96
95% CI	[−17.8, 48.3]	[−22.6, 25.8]	[4.5, 19.1]*	[1.1, 2.0]*	[−8.0, 6.2]
ρ-value	0.04*	n.s.	<0.005*	<0.005*	n.s.

*EVU*, education value unit; *R*, residency; *F*, fellowships; *S*, medical students

* significant p value
